# Tight junction proteins in glial tumors development and progression

**DOI:** 10.3389/fncel.2025.1541885

**Published:** 2025-02-03

**Authors:** Jakub Moskal, Slawomir Michalak

**Affiliations:** ^1^Department of Neurosurgery and Neurotraumatology, Poznan University of Medical Sciences, Poznan, Poland; ^2^Department of Neurochemistry and Neuropathology, Poznan University of Medical Sciences, Poznan, Poland

**Keywords:** tight junction, claudin family, TJ-associated Marvel protein family, junctional adhesion molecule family, *zonula occludens* protein, glial tumors

## Abstract

Tight junctions form a paracellular barrier in epithelial and endothelial cells, and they regulate the diffusion of fluids, molecules, and the penetration of cells across tissue compartments. Tight junctions are composed of a group of integral membrane proteins, which include the claudin family, tight junction-associated Marvel protein family, junctional adhesion molecule family, and proteins that anchor the cytoskeleton, such as *zonula occludens* proteins and the cingulin family. Several factors, such as neurotransmitters or cytokines, and processes like ischemia/hypoxia, inflammation, tumorigenesis, phosphorylation/dephosphorylation, ubiquitination, and palmitoylation, regulate tight junction proteins. Claudins are involved in tumorigenesis processes that lead to glioma formation. In gliomas, there is a noticeable dysregulation of claudins, occludin, and *zonula occludens-1* abundance, and their dislocation has been observed. The weakening of intercellular adhesion and cell detachment is responsible for glioma infiltration into surrounding tissues. Furthermore, the paracellular permeability of the blood–brain barrier, formed with the involvement of tight junction proteins, influences the development of peritumoral edema – and, simultaneously, the rate of drug delivery to the glial tumor. Understanding the junctional and paracellular environments in brain tumors is crucial to predicting glial tumor progression and the feasibility of chemotherapeutic drug delivery. This knowledge may also illuminate differences between high and low-grade gliomas.

## 1 Introduction

### 1.1 Definition of the tight junction (TJ) and the discovery of its structure and function

#### 1.1.1 TJs structure

Historically, research on transporting epithelia contributed to the discovery of intercellular structures that regulate fluxes through intercellular spaces (Farquhar and Palade, 1963). The advent of electron microscopy in the early sixties of the 20th century allowed for the visualization of membrane-associated fusions that were not previously observable with optical microscopes (Farquhar and Palade, 1963). Studies conducted on rodents revealed the intricacy of intercellular junctional structures. These parts were identified as *zonula occludens* (tight junction), *zonula adhaerens* (intermediary junction), and *macula adhaerens* (desmosome) (Farquhar and Palade, 1963). The development of the freeze-fracture method in electron microscopy by Steere (1957) laid the foundation for new possibilities in TJ research. Consequently, the next significant development in TJ studies was the application of the freeze-fracture technique (Chalcroft and Bullivant, 1970). As a result, the presence of TJs containing ridges, which interconnect membranes adjacent to gap junctions and non-junctional membranes, was finally illustrated (Chalcroft and Bullivant, 1970).

According to current knowledge, Tight Junctions (TJ) establish a paracellular barrier in epithelial and endothelial cells, regulating the diffusion of fluids, molecules, and cell penetration across tissues.

**Figure 1:**
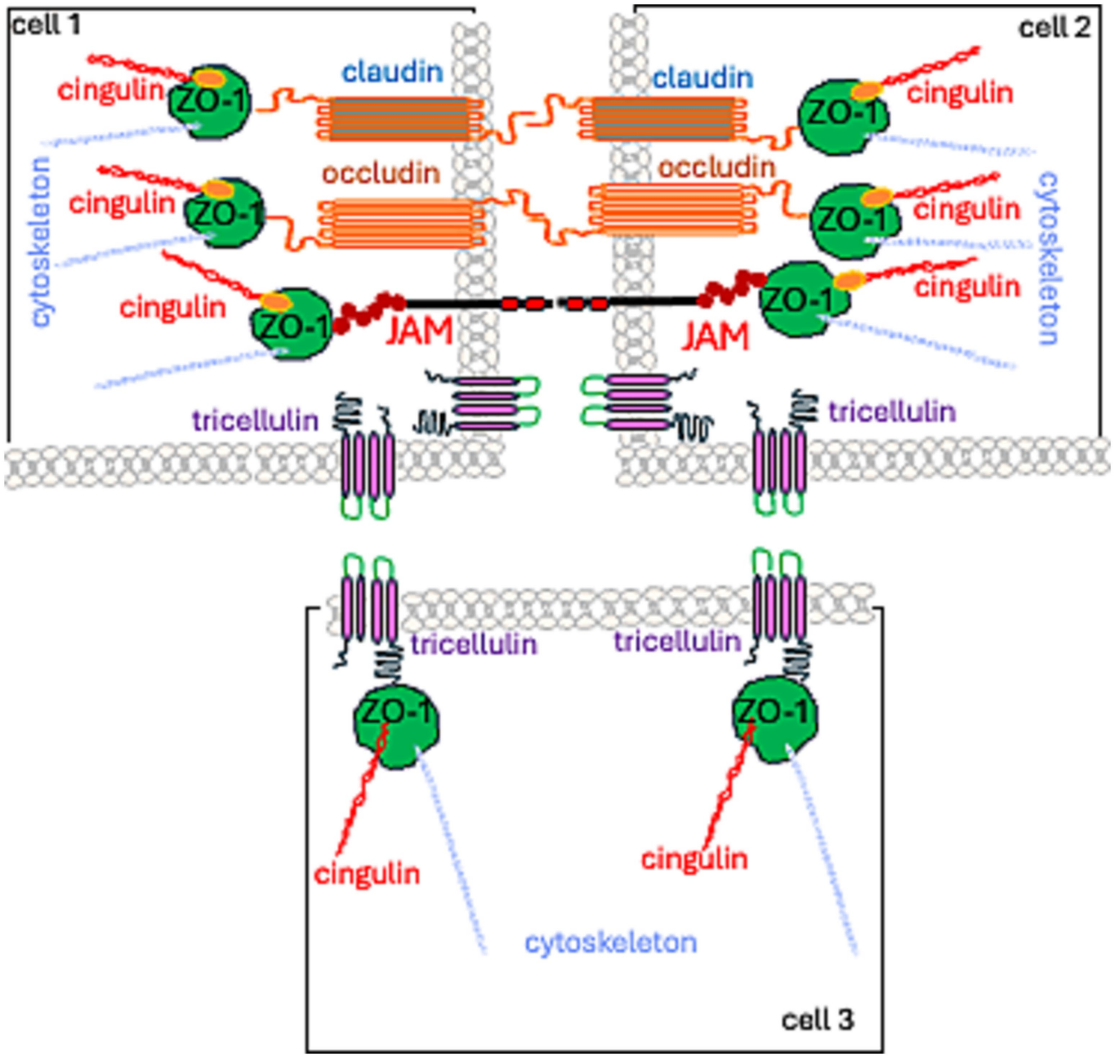
Spatial localization of TJ proteins.

TJs are formed by: (1) a group of integral membrane proteins including, the claudin family (CLDN), TJ-associated Marvel protein (TAMP) family, junctional adhesion molecule (JAM) family, and the Crumbs family of integral membrane proteins (CRBs) to regulate their function; (2) proteins that anchor the cytoskeleton: *zonula occludens* (ZO) proteins and the cingulin family (Wibbe and Ebnet, 2023; Citi et al., 2024; Figure 1).

#### 1.1.2 TJ function

The primary function of TJs stems from their location and structure, the function being fencing and gating. Fencing refers to preserving the cellular polarity, with an apical membrane domain that is structurally, molecularly, and physiologically different from the basolateral one. Gating pertains to controlling the passage of ions, molecules, and water through the paracellular pathway. This process can be detected through the measurement of the tissue’s transepithelial electrical resistance (TER).

Transepithelial/transendothelial electrical resistance (TEER) was recorded in blood–brain barrier (BBB) models (Srinivasan et al., 2015). Electrical resistance is inversely correlated with the water flux through the paracellular pathway. In brain endothelium, it was estimated to be 1870 × cm^2^, indicating a very “tight” BBB (Crone and Olesen, 1982). Claude and Goodenough made an important assumption, considering the effects of strands that form TJs as electrical resistors. Consequently, the authors demonstrated a stepwise TEER increase with the number of strands (Claude and Goodenough, 1973). Claude (1978), utilizing experimental, functional, and morphological data, performed calculations indicating that the relationship between the specific resistance of the ZO and the number of strands is non-linear. Freeze-fracture studies using electron microscopy revealed the presence of molecular strands with intramembranous units. Subsequent studies showed that the number of strands, their assembly, and TJ assembly and sealing contribute to TER (Balda et al., 1991). Factors such as the rearrangement of strands and their number (McCarthy et al., 1996), composition of occludin (OCL) and claudin (CLDN) containing strands (Tsukita and Furuse, 1999), the presence of channels and vertically oriented strands, as well as interaction with cytoskeleton and intracellular proteins (such as calmodulin, G proteins), elucidate the complex relationship between TER and strand number (Claude, 1978; Balda et al., 1996).

**Figure 2:**
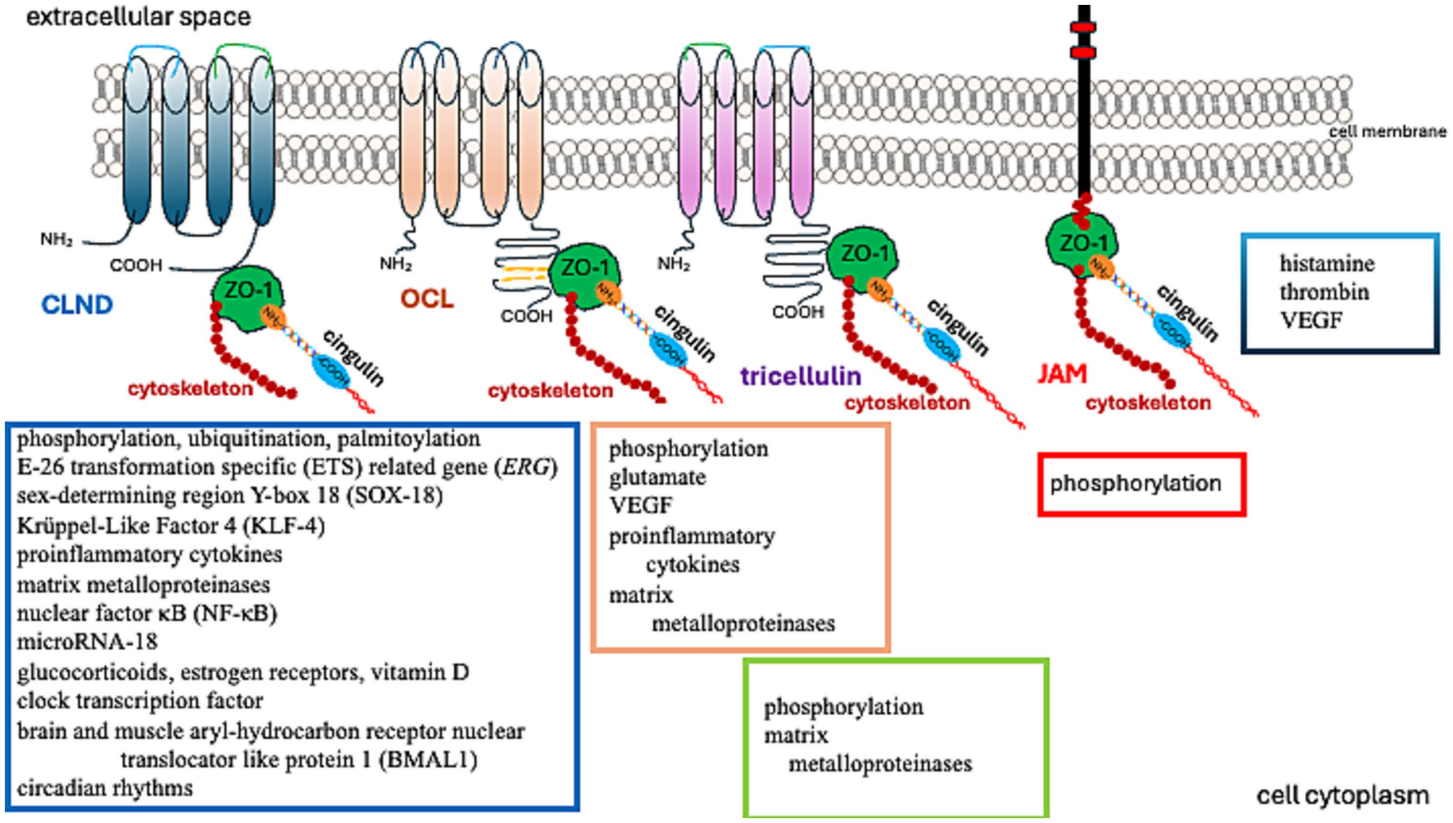
Factors regulating TJ protein function.

Currently, TJs are viewed as cell–cell adhesion complexes that act as gatekeepers in the paracellular space. Advances in research—from electron microscopy to molecular studies—have revealed that these strands are polymers of various occludin (OCL) and claudin (CLDN) proteins. They are assembled side by side (in *cis*) within the cell membrane and aligned directly (in *trans*) with proteins from neighboring cells. The resulting structure is zipper-like, closing the gap between adjacent cells (Marsch et al., 2024; Figures 1, 2).

Matrix metalloproteinases (MMPs) degrade claudin-5 (CLDN-5) in the BBB, reducing its abundance in the brain and raising its level in the cerebrospinal fluid (Chiu and Lai, 2013). OCL, another critical TJ protein, is also a substrate of MMP (Pan et al., 2021). Furthermore, MMP-2/9 cleaves ZO-1, leading to BBB disruption (Zhang et al., 2018). Thus, MMPs are involved in the process of removing TJs from their original location. Removal of proteins from the TJ and their reduced abundance can degrade the integrity of the BBB (Zheng et al., 2023).

Further studies have demonstrated that TJs are involved in regulating cell proliferation, differentiation, and apoptosis. These functions are associated with their signal transduction abilities (Matter and Balda, 2003; Matter et al., 2005). While early research primarily investigated epithelial TJs, the properties of endothelial TJs have become increasingly significant in understanding brain tumor pathomechanisms. Endothelial TJs can be influenced by various factors and conditions, as described in Table 1. Notably, amongst others, the *CLDN5* gene expression is boosted by transcriptional factors such as the E-26 Transformation Specific (*ETS*) related gene (*ERG*), ETS-1, Sex-Determining Region Y-Box 18 (*SOX-18*), Krüppel-Like Factor 4 (*KLF-4*), glucocorticoids, estrogen receptors, vitamin D, clock transcription factor, and the Brain and Muscle Aryl-hydrocarbon Receptor Nuclear Translocator Like Protein 1 (BMAL1). These can also be affected by circadian rhythms, as the expression of *Cldn5* tends to be more prominent in the mornings (Yuan et al., 2012; Fontijn et al., 2008; Ma et al., 2014a; Felinski et al., 2008; Burek et al., 2010; Zhang et al., 2022; Westgate et al., 2008).

**Table 1:** Factors, conditions, and processes that modify TJ function (Figure 2).

Factors	Conditions/activity
Neurotransmitters: glutamate Cytokines/mediators: TNFa, IL-1, VEGF Nuclear factor κB Reactive oxygen species Proteolytic enzymes – matrix metalloproteinases miRNA Toxins Drugs Nanoparticles	Ischemia/hypoxia Inflammation Tumorigenesis Phosphorylation/dephosphorylation Ubiquitination Palmitoylation

The expression of *CLDN5* is diminished by pro-inflammatory cytokines, nuclear factor κB (NF-κB), and microRNA-18, which depresses the runt-related transcription factor 1 (*RUNX1*) (Aslam et al., 2012; Miao et al., 2015).

Glutamate increases tyrosine phosphorylation and decreases threonine phosphorylation of OCL in brain microvascular endothelial cells through N-methyl-D-aspartate or alpha-amino-3-hydroxy-5-methylisoxazole-4-propionate/kainate receptors, thus disrupting the BBB (András et al., 2007). Similarly, vascular endothelial growth factor (VEGF)-induced OCL phosphorylation heightens BBB permeability (Antonetti et al., 1999).

Endothelial TJs are more sensitive to changes in the microenvironment than epithelial TJs. Several factors, conditions, and processes can modify the endothelial barrier by (1) increasing or decreasing the abundance of TJ proteins, (2) rearranging and relocating TJ molecules, and (3) posttranslationally modifying TJ proteins (Table 1). The reorganization of TJ architecture can disrupt the endothelial barrier, leading to leakage of fluids, solutes, macromolecules, and cells. This can negatively impact vascular homeostasis and brain tissue. A comprehensive understanding of TJ protein regulation presents new possibilities for intervention. In the following sections, we review current knowledge about TJ proteins (Table 2), with a particular focus on their role in primary glial tumors.

**Table 2:** TJ protein families and their representatives.

Protein family	Protein	Localization in nervous system
Claudins	Claudin-3	Brain endothelium (Wolburg et al., 2003) Oli-neu cells (a model of oligodendroglial studies) (Chen Y. et al., 2021)
	Claudin-5	Brain endothelium (Hashimoto et al., 2023)
	Claudin-11	CNS myelin/oligodendrocytes (Bronstein et al., 2000; Gjervan et al., 2024)
	Claudin-12	Brain endothelium (Vanlandewijck et al., 2018; Garcia et al., 2022) Oligodendrocytes, astrocytes, neurons (Castro Dias et al., 2019)
	Claudin-25	Brain endothelium Oligodendrocytes (Vanlandewijck et al., 2018; Garcia et al., 2022)
TAMP family [tight junction- associated MARVEL (MAL and related proteins for vesicle trafficking and membrane link) protein]	Occludin	Brain endothelium pericytes Astrocytes (Castro et al., 2018) Neurons (Bauer et al., 1999)
	Tricellulin (MARVEL D2)	Brain endothelium (Iwamoto et al., 2014) Astrocytes neurons (Mariano et al., 2013) Microglia (Kojima et al., 2013)
	MARVEL D3	Brain endothelium (Tornabene et al., 2019)
ZO	*Zonula occudens* – 1, −2, −3	Brain endothelium (Gray et al., 2019) Astrocytes (Morgan, 2016)
JAMs	Junctional Adhesion Molecules	Brain endothelium (Kakogiannos et al., 2020)
Cingulin		Brain endothelium (Schossleitner et al., 2016)

#### 1.1.3 TJ proteins

##### 1.1.3.1 *Claudins* (CLDNs)

**Figure 3:**
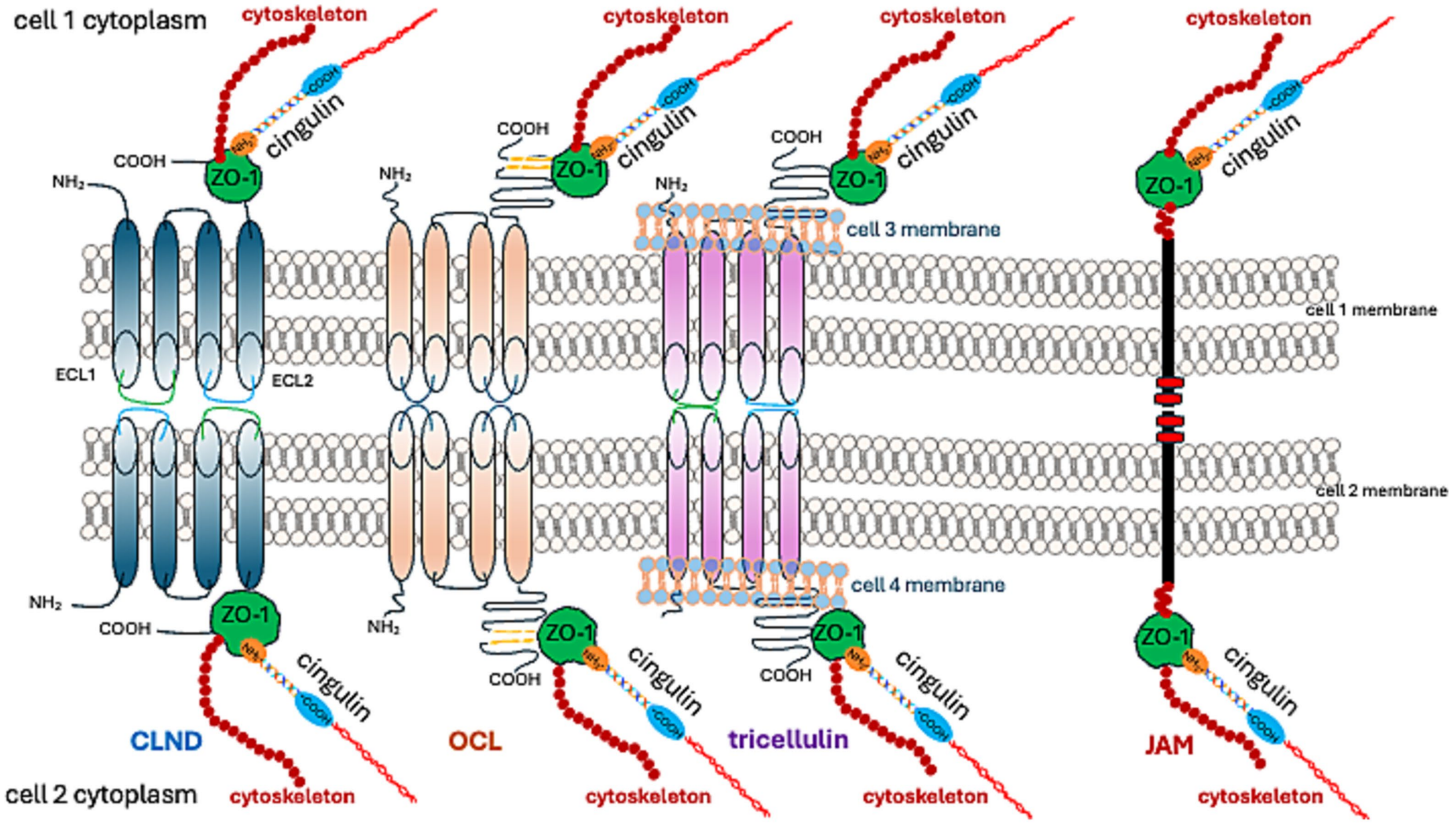
TJ proteins and their interactions at cell–cell adhesion sites.

Claudins (CLDNs) constitute a family of 27 proteins, categorized based on their configuration in TJs’ meshwork into class A, forming large mesh sizes (CLDN-7, CLDN-10a with anion channel attributes, CLDN-19a/b, −20), class B generating small mesh sizes (cation channel-forming CLDN-2, −10b, −15, and barrier-forming CLDN-3, −5, −14), and class C (CLDN-1, −6, −9, −11), shaping a tightly packed meshwork with parallel strands (Gonschior et al., 2022). From an amino acid sequence perspective, CLDNs are typified as “classic” and “non-classic.” “Classic” CLDNs are CLDN-1 to 10, −14, −15, −17, −19, which build strands between neighboring cells via trans-interactions (Hashimoto et al., 2023) and share sequence homology (Figure 3). The remaining CLDNs are defined as “non-classic.”

CLDN-1, −3, −5, −11, −18, and − 19 form a barrier that excludes ion and solute flux. CLDN-2, −4, −8, −10, −15, −16, −17, and − 21 form ion channels. The function of other CLDNs depends on their cooperation with other proteins (Hashimoto et al., 2023).

The palmitoylation of CLDN-5 increases its affinity for cholesterol-rich domains within membranes, inhibits its self-assembly, and stabilizes TJ proteins within a cholesterol-rich membrane (Rajagopal et al., 2019). The degradation of CLDN-5, along with other CLDNs, is regulated by the ubiquitin-proteasome pathway (Mandel et al., 2012; Takahashi et al., 2009).

While TJs in endothelial cells are primarily composed of CLDN-5, they also include occludin (OCL), tricellulin, JAMs, and angulin-1 (lipolysis-stimulating receptor; LSR) (Vanlandewijck et al., 2018). The preservation of TJ structure and functionality necessitates binding to ZO-1 (Vasileva et al., 2022; Sugawara et al., 2021; Li et al., 2005; Itoh et al., 1999).

##### 1.1.3.2 TAMPs

The TAMP (tight junction-associated MARVEL [(MAL {myelin and lymphocyte} and related proteins for vesicle trafficking and membrane link] protein) family comprises occludin, tricellulin (MARVEL D2), and MARVEL D3. MARVEL is a domain formed by four transmembrane-helix molecules identified in MAL proteins, physins, gyrins, and OCL (Sánchez-Pulido et al., 2002).

OCL was the first identified TJ protein (Furuse et al., 1993). Initially, it was discovered in avian tissues; nonetheless, later comprehensive studies revealed that it is also expressed in mammalian tissues. The NH-2 terminal half of OCL contains four membrane-spanning domains. The C-terminal portion of OCL is crucial for its interactions with ZO-1 protein dimerization and signaling function (Ando-Akatsuka et al., 1996; Li et al., 2005; Walter et al., 2009a; Walter et al., 2009b).

Five domains (A to E) arise from the transmembrane localization of OCL (Furuse et al., 1994). Domains A to D are found in the NH2-terminal, while domain E is present in the COOH-terminal half (Furuse et al., 1994). The release of this factor into the bloodstream suggests it could serve as a biomarker of BBB disintegration, having been observed in various brain pathologies (Kazmierski et al., 2012). Additionally, two forms of OCL have been identified: low-molecular-weight (HWM) (65 kDa to 68 kDa) and high-molecular-weight (LWM) (72 kDa to 75 kDa and 70 kDa to 7 kDa) (Wong, 1997). They are related to different TER; LWM associates with a lower TER (approximately 30 × cm^2^), while HMW associates with a higher TER (approximately 3,300 × cm^2^) (Wong, 1997).

OCL function is moderated by serine/threonine phosphorylation and tyrosine by different kinases. HMW OCL is more likely to be hyperphosphorylated than LMW (Wong, 1997). The distribution and function of OCL rely upon phosphorylation, with hyperphosphorylated HMW forms accumulating at cell–cell junctions, indicative of the presence of OCL’s functional forms at TJ (Wong and Gumbiner, 1997). Protein kinase C (*PKC*) plays a part in OCL phosphorylation (Tai et al., 1996). Activating PKC enhances OCL’s abundance in TJ via mitogen-activated protein kinase (MAPK), a step correlated with a decrease in TER (Wang et al., 2004). PKC stimulators like phorbol 12-myristate 13-acetate (PMA) and 1,2-dioctanoylglycerol affect OCL’s phosphorylation and distribution (Andreeva et al., 2001).

Conversely, dephosphorylation of serine/threonine residues on OCL by protein phosphatase 2A (PP2A) leads to TJ disintegration and increased paracellular permeability (Nunbhakdi-Craig et al., 2002). Regulating the phosphorylation status of OCL is phospholipase C-*γ* (PLC-γ) (Nunbhakdi-Craig et al., 2002), which contributes to hydrolyzing phosphatidylinositol 4,5-bisphosphate (PIP 2) to produce diacylglycerol (DAG) and inositol 1,4,5-trisphosphate (IP3). IP3 provokes a transient increase of free intracellular Ca2+, while DAG directly activates PKC (Berridge and Irvine, 1989; Nishizuka, 1986).

Inhibition of phospholipase Cγ results in hyperphosphorylation of ZO-1, ZO2, and OCL correlating with TEER reduction (Ward et al., 2002). Also, PMA-induced PKC activation influences OCL dephosphorylation (Clarke et al., 2000). This effect may result from the inactivation of a serine/threonine kinase or the activation of a serine/threonine phosphatase. Therefore, PKC regulates signaling pathways leading to a decline in phosphorylated OCL and increased TJ permeability (Clarke et al., 2000). These findings highlight the complexity of OCL regulation and the involvement of other intermediaries in the pathways linking PKC and OCL.

Among other regulators of intracellular processes, small GTPases play a significant role. These enzymes catalyze the hydrolysis of guanosine triphosphate (GTP) to guanosine diphosphate (GDP) and represent a class of GTP-binding proteins known as the Ras superfamily, comprising of Rho, Rab, Arf, and Rab. They regulate the structure and function of OCL. Notably, RhoA and Rac1 are involved in this process (Jou et al., 1998).

Mitogen-activated protein kinases (MAP kinases) belong to another family of Ser/Thr kinases (ERK, p38/MAPK, and JNK). These kinases are activated by a sequence of kinases (MAPK kinase or MEK; MAPKK kinase) that help mediate the protective effects of epidermal growth factor (EGF) on TJs. EGF stabilizes TJ against H_2_O_2_-induced disruption by inhibiting H_2_O_2_-induced Tyr-phosphorylation, Thr-dephosphorylation, and cellular redistribution of OCL and ZO-1 (Basuroy et al., 2006). The protective effect of EGF against oxidative stress plays a key role in the progression of glioblastoma. Macrophages and glioblastoma-surrounding microglia secrete EGF, which takes part in the interplay of tumor-associated macrophage (TAM) and glioma cells, contributing to an environment that supports tumor progression (Buonfiglioli and Hambardzumyan, 2021). Oxidative stress results in OCL phosphorylation by tyrosine kinase, leading to the disassembly of the OCL-ZO complex (Rao et al., 1997) because C-terminal phosphorylation weakens the binding ability of OCL to ZO proteins (Kale et al., 2003). The combination of oxidative stress and inflammation, resulting from activated macrophages in the glioblastoma microenvironment, plays a role in the development and progress of the tumor (Xing et al., 2024). Exposing cell cultures to pro-inflammatory cytokines IFNγ and TNF*α* increases OCL abundance and TER (Van Itallie et al., 2010). The component linking inflammation and glioblastoma progression is the Src family of kinases (SFKs). An increased abundance of SFKs is seen in tumor-infiltrating cells and tumor cells (Zhao et al., 2024a; Torrisi et al., 2020), which catalyzes OCL phosphorylation (Kale et al., 2003). OCL stability is affected by the protein tyrosine phosphatase inhibitor phenylarsine oxide (PAO), alongside another inhibitor – pervanadate (PV), resulting in a TER decrease (Lohmann et al., 2004). PAO’s effect is activated by matrix metalloproteinase (Lohmann et al., 2004), while PV’s effect is not. Thus, the complex control of OCL through tyrosine phosphorylation, similar to serine/threonine phosphorylation, involves various undetermined factors. The presence of phosphatidylinositol (PI) 3-kinase (PI3K), which is associated with activated growth factor receptor tyrosine kinases, interacts with the coiled-coil domain of OCL (Nusrat et al., 2000).

OCL may impact glucose transport and metabolism. It co-localizes with insulin-responsive glucose transporters GLUT4 and GLUT1 on the BBB endothelial cells (Ngarmukos et al., 2001). Furthermore, OCL’s role also hinges on the downstream enzymatic activity of NADH oxidase, which relies on the NADH binding site in the CC-domain binding site, converting NADH to NAD+ (Torrisi et al., 2020). OCL activates the nuclear NAD + -dependent histone deacetylase sirtuin-1 (SIRT-1) (Castro et al., 2018). It governs glucose uptake and ATP production by elevating the activity of AMP-activated protein kinase (Castro et al., 2018). Changes in OCL abundance correlate with glucose transporters (Castro et al., 2018).

As previously stated, the C-terminal fragment of OCL binds to the cytoplasmic ZO-1 protein. ZO proteins (ZO-1, ZO-2, ZO-3) comprise a family of cytoplasmic proteins that not only connect to the actin cytoskeleton (Figures 1, 3) but also to CLDNs and actin-binding proteins such as *α*-catenin and cortactin (Katsube et al., 1998). ZOs also belong to the membrane-associated guanylate kinase homolog (MAGuK) protein family and form cytosolic plaques at TJs (Anderson et al., 1995). They are the most pivotal cytosolic TJ proteins due to their scaffolding functions via multiple binding sites, regulation of cytoskeletal organization, establishment of cell polarity, and signaling reciprocity between the cytoplasm and the nucleus (Guillemot et al., 2008; Gottardi et al., 1996). ZO-1 undergoes phosphorylation by protein kinase C delta (PKCδ), along with CLDN-5 and OCL (Jiao et al., 2011). The phosphorylation of tyrosine residues in cerebral vessels post interleukin-1β administration was linked with a loss of TJ proteins, specifically ZO-1, and OCL (Bolton et al., 1998). Moreover, pro-inflammatory cytokines, TNF-α and IL-6, lead to a decrease in endothelial ZO-1 abundance, which is associated with phosphorylation at threonine and tyrosine residues, NADPH oxidase activation, and oxidative stress (Rochfort and Cummins, 2015).

##### 1.1.3.3 JAMs

JAMs are part of the immunoglobulin superfamily that contribute to the processes of TJ assembly and integrity (Martìn-Padura et al., 1998; Ebnet, 2017). There are three recognized members of the JAMs family; all JAMs interact with ZO-1 (Figures 1, 3) and contain two extracellular immunoglobulin-like domains. JAM-A, in particular, controls the localization of ZO-1 within TJs and as the predominant isoform in the brain endothelium, it helps govern BBB permeability (Aurrand-Lions et al., 2001). JAMs form a barrier even in the absence of CLDNs (Otani et al., 2019). Endothelial JAMs undergo phosphorylation via PKC, a process stimulated by thrombin and collagen (Ozaki et al., 2000). RhoA and Rho kinases also phosphorylate JAM-A in the brain endothelium after exposure to the CCL2 chemokine ([C-C motif] ligand 2), leading to its relocation to the TJ (Stamatovic et al., 2012). Lastly, JAM-A’s role also includes stimulating transcription factor C/EBP-α (CCAAT [cytosine-cytosine-adenosine-adenosine-thymidine] box motif/enhancer binding proteins) to increase the abundance of CLDN-5 (Kakogiannos et al., 2020).

##### 1.1.3.4 Cingulin

Cingulin was first identified as a component of an avian TJ (Citi et al., 1988), and was later located in the brain endothelium (Schossleitner et al., 2016). Cingulin can also form complexes with ZO-1. An increase in cingulin levels leads to a reduction in CLDN-5 dominance in endothelial cells (Schossleitner et al., 2016; Figure 3). Cingulin is generally present in the cytoplasm, where it forms a plaque-like structure that operates as a foundation for guanine nucleotide exchange factors (GEFs) Rho-GEF: GEF-H1, and p114RhoGEF. These enable endothelial stability (Birukova et al., 2006). The activity of Rho family GTPase at TJ, regulated by cingulin, is facilitated by a direct interaction with the GEFs: RhoA and Rac1 (Citi et al., 2012). The pro-inflammatory cytokine, TNF alpha, stimulates RhoB activation through ArhGEF10 (Rho Guanine Nucleotide Exchange Factor 10). This suggests that cingulin influences the effects of this pro-inflammatory cytokine on TJ disruption (Khan et al., 2021). Additionally, it contributes to the tumor microenvironment milieu, which is involved in controlling glioblastoma progression. Conversely, the stimulation of cingulin by inflammation mediators, such as histamine, thrombin, and VEGF, reinforces barrier integrity. This is evidenced by the increase in TEER (Holzner et al., 2021). The same study also reported cingulin phosphorylation.

The ever-growing knowledge of the structure of TJ proteins, their functions, and the mechanisms of their regulatory processes have revealed their role in the development and advancement of glial tumors. The abundance of these proteins responds to various stimuli, each associated with cell proliferation, migration, and differentiation, which are vital for carcinogenesis, tumor progression, and metastases (Martin, 2014; Leech et al., 2015; Díaz-Coránguez et al., 2019).

Thus, with this approach in mind, we subsequently discuss how insights from TJ studies can illuminate the primary TJ protein’s role in glioma formation and progression.

## 2 Tight junction proteins in glioma formation

Similar to other malignancies, gliomas are characterized by reduced cell–cell adhesion, increased cellular motility, modified metabolism, enhanced proliferation, and neovascularization. Each of these stages is crucial in the development of an aggressive malignant phenotype in glioma, a process in which the involvement of TJ proteins has been evidenced.

### 2.1 TJ role in cell–cell adhesion

The dynamic regulation of cell adhesion during the early phase is diminished, facilitating the detachment of glioma cells from the extracellular matrix (ECM). This process is coordinated by cell adhesion molecules (CD44), the neural cell adhesion molecule (NCAM), and cadherin (Edvardsen et al., 2000; Cavallaro and Christofori, 2004). Subsequently, the detached cells migrate from the primary tumor to another site by forming and breaking integrin-mediated ECM interactions (Wang et al., 2022).

The role of TJ proteins requires further extensive study, although there is some existing evidence for their involvement in tumorigenesis and proliferation. OCL binds to the Rab11 (Ras [Rat sarcoma virus] related protein) GTPase interacting protein FIP5 (Rab11 family-interacting protein 5) (Zhang et al., 2024). FIP5 is a key regulator of endocytic transport (Jordens et al., 2005), which also oversees cell migration. The Rip11/FIP5 complex is involved in perinuclear endosome recycling (Schonteich et al., 2008). The binding of OCL to this complex regulates mitotic spindle orientation and controls prophase and cell proliferation (Zhang et al., 2024). Experiments in the latter study indicate that OCL triggers the TJ disruption associated with oncogenesis (Li and Mrsny, 2000). The loss of intercellular contact corresponds to the diminished presence of OCL and CLDN-1 and the redistribution of ZO-1 and E-cadherin (Li et al., 2000).

The composition of the cellular membrane and cholesterol content was suggested to be an independent mechanism that controls TJ structure and cell–cell interactions. The assembly of CLDNs into TJ strands depends on their affinity for cholesterol-rich membrane domains (Shigetomi et al., 2018). Additionally, the ZO-1 protein contributes to TJ formation from cholesterol-rich membrane domains, acting as a scaffold for CLDNs (Shigetomi et al., 2023).

*In vitro*, cholesterol depletion in the plasma membrane reduces OCL, CLDN-2, −3, and − 7 content (Casas et al., 2010). Glioblastoma cells regulate cholesterol synthesis through sterol regulatory element-binding protein 2 (SREBP2), glucose transporters (GLUTs), and members of the Ras-related protein Rab11 (Rab11) small GTPase family (Rab11s). SREBP2 activates genes encoding enzymes involved in cholesterol synthesis; for example, 3-hydroxy-3-methylglutaric acid (HMG)-coenzyme A (CoA)-reductase (HMGCR).

The passage of cholesterol through the BBB is limited, so cholesterol is synthesized in astrocytes and this process depends on the cellular glucose content. The transport of glucose into cells is facilitated by GLUTs, membrane proteins whose function is regulated by their distribution and the trafficking of intracellular vesicles. The recycling of GLUT-containing vesicles and their fusion with the plasma membrane is regulated by Rab11 family proteins (Wieman et al., 2007). An increase in the abundance of SREBP2, Rab11s, and GLUTs is associated with poor survival in glioblastoma patients (Cheng et al., 2022).

A combination of cholesterol synthesis regulators and GLUTs increases glioblastoma proliferation and aggressiveness. Conversely, when cholesterol in the plasma membrane is depleted, TJ proteins are shed from the membrane into extracellular space, leading to proteolysis catalyzed by metalloproteinases (Casas et al., 2010). Cholesterol metabolism in glioblastoma cells produces dihydroandrosterone, hydroxytestosterone, androstenediol, androstenedione, and progesterone, which contribute to tumor progression and are considered as potential therapeutic targets (Pinacho-Garcia et al., 2020).

Finasteride and dutasteride, which inhibit the conversion of cholesterol into neurosteroids by glioblastoma cell lines, limit tumor cell proliferation (Pinacho-Garcia et al., 2020; Kim et al., 2021). Additionally, finasteride has an antioxidant effect through the activation of antioxidant genes *SESTRIN2* and *PRDX5* (PEROXIREDOXIN-5) (Kim et al., 2021), leading to a reduction in reactive oxygen species (ROS). This effect is associated with a decrease in *β*-catenin accumulation in glioblastoma cells (Kim et al., 2021). The translocation of β-catenin to the nucleus mediates the transcription of genes, including those involved in endothelial proliferation, and decreases OCL and CLDN-5 abundance, which are crucial for BBB integrity (Manukjan et al., 2024). Thus, finasteride reduces β-catenin, reverses its effect, and restores BBB integrity.

β-catenin is part of the WNT (Wingless-related integration site)/β-catenin signaling pathway that is active in glioblastoma and is considered a therapeutic target (Yun et al., 2023). Furthermore, stimulation of the WNT/β-catenin pathway promotes the epithelial-to-mesenchymal transition (EMT).

### 2.2 TJ participation in epithelial-to-mesenchymal transition (EMT)

EMT, referring to the process where an epithelial cell undergoes transcriptional, biochemical, and structural changes, was identified in glioblastoma (Yang et al., 2021) and is known to enhance tumor cell motility (Kahlert et al., 2012). The impact of EMT is characterized by the adoption of a mesenchymal cell phenotype, which is associated with the disintegration and reorganization of TJ (Kyuno et al., 2021; He et al., 2023). The EMT process has been linked with the suppression of TJ proteins such as OCL and CLDNs. The pro-inflammatory cytokine, transforming growth factor beta (TGFb), known to contribute to glioblastoma progression, is found to decrease the abundance of CLDN-4 and stimulate the hypermethylation of its gene (*CLDN4*) during EMT (Papageorgis et al., 2010). The tumor-derived TGFb2 activates matrix metalloproteinases, leading to a decrease in the abundance of OCL, CLDN-1, and CLDN-5 (Ishihara et al., 2008). Interestingly, TGFb stimulates an increase in the abundance of CLDN-3 in glioblastoma, with a higher content of this TJ protein associated with EMT stimulation. Reciprocally, CLDN-3 has been found to enhance the effects of TGF-β (Sun et al., 2023).

The abundance of CLDN-4, which plays a pivotal role in tumor progression, is increased in glioblastoma (Yan et al., 2022). The involvement of TJ proteins in EMT, however, is complex and depends on their specific location and activity. CLDNs foster the activation of matrix metalloproteinases necessary for EMT (Miyamori et al., 2001). ZO-1 oscillates between the membrane, cytoplasm, or nucleus, and its localization in the nucleus is linked with tumor invasiveness (Polette et al., 2007). Depending on their location and cancer type, TJ proteins can either promote or suppress the EMT of cancer cells (Kyuno et al., 2021). Such diversity has also been observed in glial tumors and is related to cancerogenesis pathways like the Switch/Sucrose Non-Fermentable (SWI/SNF) chromatin remodeling complex. SWI/SNF pries open the chromatin and aid in modulating its structure through the energy derived from ATP hydrolysis. This results in disrupted histone-DNA binding and increased DNA availability for transcription factors to modulate gene expression and DNA repair. Generally considered tumor suppressors, *SWI/SNF* genes account for mutations in almost 20% of all human cancers (Kadoch et al., 2013). Studies on CLDN-4 abundance in SWI/SNF-deficient neoplasms indicate that this complex lowers CLDN-4 levels (Schaefer et al., 2017). Furthermore, in H3.3K27M diffuse intrinsic pontine gliomas, there is an elevated presence of the SWI/SNF complex ATPase subunits, SMARCA4 and SMARCA2 (Switch/Sucrose Non-Fermentable (SWI/SNF)-Related, Matrix-Associated, Actin-Dependent Regulator of Chromatin, Subfamily A) (Mota et al., 2023). As such, a correlation between SWI/SNF mutation and an increase in CLDN-4 abundance is likely in certain types of gliomas. Glioblastoma cells also show variations in other CLDNs, such as increased levels of CLDN-1 and decreased levels of CLDN-5 (Schweiger and Kievit, 2023).

### 2.3 Neovascularization in glioma and TJ proteins

Neovascularization is another process in which TJ proteins participate. Vascular hyperplasia is a common histological feature of glioblastoma and is stimulated by the activation of the HIF-1 pathway (Hypoxia-inducible factor − 1). HIF-1, a key transcription factor mediating the hypoxia response, the levels of which increase in gliomas, causes ZO-1 dysregulation (Lin and Wu, 2023). The rise in HIF-1 abundance correlates with the loss of ZO-1 and increased paracellular permeability in the endothelium (Meng et al., 2019). Endothelial cells associated with glioblastoma are resistant to apoptosis and cytotoxic agents, exhibit heightened motility, and produce substantial amounts of growth factors like VEGF. Five clusters of endothelial cells were identified in glioblastoma (Xie et al., 2021). Cluster 1 of endothelial cells originated from nonmalignant brain tissue surrounding the glioblastoma and was characterized by high expression of *KLF2 (Krüppel-like family of transcription factor 2*), *TIMP3* (*tissue inhibitor 3 of metalloproteinases*), *SLC2A1* (*solute carrier family 2 member 1* encoding glucose transporter protein type 1, [GLUT1])*, SLCO1A2 (solute carrier organic anion transporter family member 1A2*), and highest expression of *CLDN-5*, high *OCL*, and *JAM2* (Xie et al., 2021). OCL, CLDN-3, and CLDN-5 were observed in most glioblastoma microvessels at levels akin to normal brain tissue (Wolburg et al., 2003). However, some vessels showed decreased OCL abundance or its presence was observed outside TJ. A fraction of glioblastoma microvessels demonstrated a total loss of CLDN-3 and CLDN-5 (Wolburg et al., 2003). The diversity observed in various subsets of microvessels arises from features reported in five endothelial cell clusters. A recent co-culture model of endothelial cells with glioblastoma spheroids showed a decreased content of OCL and CLDN-5 (Mathew-Schmitt et al., 2024). Most glioblastoma microvessels showed a total loss of CLDN-1, while hyperplastic vessels exhibited a decreased abundance of CLDN-5 and OCL (Liebner et al., 2000).

### 2.4 The role of cell interactions and tight junction dynamics in glioblastoma progression

The crosstalk between glioblastoma cells and endothelial cells within the tumor microenvironment significantly impacts tumor progression and prognosis. Agrin, a basement membrane heparan sulfate proteoglycan, plays a crucial role in this interaction by promoting tumor angiogenesis (Steiner et al., 2014). It increases the abundance of normal brain endothelial (VE)-cadherin, *β*-catenin, and ZO-1, but does not affect CLDN-5 and OCL (Steiner et al., 2014). In glioblastoma microvessels, agrin’s absence correlates with diminution in CLDN-1, CLDN-5, and OCL. Conversely, when agrin is present, it is accompanied by an increase in OCL and CLDN-5 but not in CLDN-1. Furthermore, agrin deficiency leads to an increase in the tenascin content within the glioblastoma ECM (Rascher et al., 2002).

The Glial cell line-derived neurotrophic factor (GDNF), secreted by astrocytes, enhances the abundance of CLDN-5, OCL, and ZO-1 in the endothelium of tumor vessels, which helps protect the BBB (Igarashi et al., 1999; Yue and Hoi, 2023; Liu T. et al., 2022). Likewise, GDNF boosts the presence of CLDN-5 and VE-cadherin in neurons (Yang et al., 2024). Intriguingly, a recent study noted that GDNF, apart from the prior effect, quickens the migration and invasion of glioblastoma cells by raising the quantity of the serine protease inhibitor family E member (SERPINE)-1 through signaling via the small mothers against decapentaplegic (SMAD) homolog 2/3 pathway (Guo et al., 2024).

Inflammatory cytokines enhance the quantity of CLDN-1, CLDN-4, and JAM-A in astrocytes, forming a layer consisting of astrocytic end-feet processes that envelop brain microvessels. This creates a secondary barrier in response to lesion formation (Horng et al., 2017). Specific impacts of cytokines on TJ proteins have been detected, with IL-1*β*, IFN-*γ*, and TGF-β1 all inducing CLDN-1, and IL-1β and TGF-β1 inducing CLDN-4 and JAM-A (Horng et al., 2017). IL-1β reduces OCL abundance without impacting ZO-1 and ZO-2 abundance, significantly altering astrocyte-to-astrocyte connectivity (Duffy et al., 2000).

Perivascular end-feet of reactive astrocytes consist of CLDN-1, CLDN-4, and JAM-A, whereas the loss of endothelial CLDN-5 was observed in inflammatory lesions (Horng et al., 2017). More so, activated lymphocytes cluster together when co-cultured with IL-1β stimulated astrocytes, a process limited by silencing of CLDN-1, CLDN-4, or JAM-A. Activated T lymphocytes penetrate the BBB due to their production of serine proteases and MMPs, primarily degrading CLDN-4 and, to a lesser extent, CLDN-1.

Reactive astrocytes transmit inflammation in a BBB model through a TNF-STAT3 signaling axis, and the secretion of alpha 1-antichymotrypsin regulated by the *SERPINA3* gene (Kim et al., 2022). Consequently, the abundance of CLDN-5 reduces. The disassembly of tricellular junctions (Figure 1) is suggested to augment transcellular lymphocyte T diapedesis, stimulated by the presence of chemokines on the outer wall of the tumor vasculature (Castro Dias et al., 2021). Thus, astrocyte-dependent regulations of TJ proteins through inflammatory reactions create an immunological milieu that dictates whether the immune system resists or tolerates glioma tumorigenesis (Kumari et al., 2024).

Pro-inflammatory cytokines alter TJ proteins leading to BBB disintegration. Endothelial cells are crucial in maintaining BBB integrity through tight junctions, while astrocytes supply and sustain TJ proteins—like CLDN-5—in brain endothelial cells (Hashimoto et al., 2023). Therefore, the disassembly of TJs and the subsequent functional impairment of the BBB accompany glioblastoma tumor progression (On et al., 2013).

## 3 Tight junction proteins in glioma progression

Once a glial tumor invades the brain parenchyma, it cannot be eradicated surgically, and its recurrence cannot be prevented. Invasion is an orchestrated process involving the detachment of tumor cells from the central mass, migration through the brain parenchyma, and reattachment at a new site. Both cell-to-cell and cell-to-ECM adhesion significantly influence glioma invasion. As mediators of intercellular adhesion, TJ proteins are critical players in tumor cell proliferation, neovascularization, and glioma progression.

### 3.1 Procarcinogenic pathways in glioma and TJ proteins

EGF and its receptor (EGFR) are known to play a significant role in the development of glioblastoma, as *EGFR* gene modifications, like amplification, point mutations, deletions, or hypermethylation, have been identified in the tumor (Saadeh et al., 2018). EGFR triggers the activation of MAPKs, which involve mitogen-activated extracellular kinase/extracellular signal-regulated kinases (MEK1/2-ERK1/2) participating in the phosphorylation of the ETS (E 26) domain-containing protein-1 (ELK1) (Matsuoka et al., 2022). Furthermore, EGF-induced ELK1 phosphorylation increases CLDND1 expression at the mRNA level and enhances the abundance of this TJ protein (Matsuoka et al., 2022).

The TGF-β/CLDN4/TNF-*α*/NF-*κ*B signaling axis plays a pivotal role in the biological progression of glioma, involving CLDN-4. Its suppression curtails mesenchymal transformation, cell invasion, migration, and glioma growth. TGF-β elevates CLDN-4 abundance, facilitating glioblastoma cell invasion. Furthermore, CLDN-4 stimulates TNF-α and NF-κ signaling. CLDN-4 escalates the expression of mesenchymal-related genes in glioblastoma and augments its mesenchymal transition and invasion capability (Yan et al., 2022). CLDN-4 also catalyzes Wnt3A, a pathway crucial for glioma progression. Wnt3A, a derivative of the *WNT* gene, regulates self-renewal and differentiation in the central nervous system. Neurontin (NNAT), a proteolipid governing overall body metabolism, mediates the CLDN-4 effect on Wnt3A, leading to glioma progression (Yang et al., 2023). Expression of the *WNT5A* gene is heightened in low-grade glioma and glioblastoma, correlating with poor prognosis in low-grade glioma (Feng et al., 2022). The WNT5a protein is a key effector in the CUX1 (CUT-like homeobox 1)/WNT5a/NFAT (nuclear factor of activated T cells) axis and increased CUX1 abundance is associated with poor prognosis in glioma (Feng et al., 2021). The p75 isoform of the CUX1 protein, also identified as the CCAAT displacement protein (CDP), reduces CLDN, ZO-1, and E-cadherin in glioma cells, promoting tumor infiltration capabilities (Xu et al., 2021).

### 3.2 MicroRNAs and TJ proteins

MicroRNAs, which affect a multitude of processes involved in carcinogenesis, contribute to the pathogenesis of glial tumors, and their impact on TJs’ proteins is significant (Beylerli et al., 2022; Ordóñez-Rubiano et al., 2024). MicroRNA carriers are exosomes, a 30–100 nm subset of extracellular vesicles (EVs) that are shed from cells as membrane-coated particles containing cytoplasmic or membrane components (Ghaemmaghami et al., 2020). In addition to microRNA, exosomes transport proteins, lipids, and DNAs, playing a crucial role in intercellular communication. Exosomes can be secreted by both glioblastoma and mesenchymal stem cells. Hypoxic glioblastoma cells excrete exosomes containing miR-301a, targeting the tumor suppressor gene TCEAL7 via the Wnt/b-catenin pathway, leading to a tumor resistant to radiotherapy (Yue et al., 2019). Exosomes containing microRNAs are emerging as promising biomarkers for indicating glioma progression and treatment response (Yin et al., 2019; Zeng et al., 2018). Analyzing exosome content to represent the molecular phenotype of glioma cells is a novel method in oncological testing known as “liquid biopsy,” reviewed extensively elsewhere (Ghaemmaghami et al., 2020).

The long noncoding RNA nuclear paraspeckle assembly transcript 1 (lncRNA NEAT1) associates with the miR-181d-5p/SOX5 pathway, leading to decreased OCL, CLDN-5, and ZO-1 abundance and increased BBB permeability (Guo et al., 2017). The lncRNAs regulate aspects such as cell-cycle regulation, cell development, migration, and apoptosis (Zhang et al., 2019). An lncRNA NEAT1 isoform imbalance has recently been identified as a cause of transcriptomic changes in glioma (Zakutansky et al., 2024). The downstream effect of lncRNA NEAT1 is related to microRNA miR-181d, which inhibits methyl-guanine-methyltransferase (MGMT), an effect that correlates with a better response to temozolomide and extended overall survival (Zhang et al., 2012).

MicroRNAs (miRNAs) are noncoding short RNAs (18–22 nucleotides). Their precursors are either miRNA genes or coding gene introns subject to RNA polymerase II modification. MicroRNAs bind to the 3′ untranslated terminal (3′ UTR) mRNA areas of the target gene and inhibit its translation, inducing a silencing effect (Shang et al., 2023). They take part in regulating junction protein gene expression and thereby control TJ structure integrity (Zhuang et al., 2016). Experimental models in endothelial cells have shown that miR-98 decreases ZO-1 abundance without affecting OCL and CLDN-1 while increasing the factor-inhibiting HIF-1 (FIH-1) level and reducing hypoxia-inducible factor-1 (HIF-1*α*) abundance (Hu et al., 2015).

HIF-1 abundance has been shown to correlate with glioblastoma progression, neovascularization, glucose metabolism, migration, invasion, and patient survival (Huang et al., 2019). MicroRNAs regulate HIF-1 effects in astrocytoma and glioblastoma. For example, MiR2243p negatively regulates HIF-1 abundance in glial tumor cells (Huang et al., 2019). MicroRNAs such as miR-181a decrease OCL, CLDN-5, and ZO-1 abundance and increase permeability in the co-culture of glioma cells with endothelial cells through a process meditated by Krüppel-like factor 6 (KLF6), a transcription factor that interacts with the promoters of TJ proteins genes (Ma et al., 2014b).

In a glioma blood-tumor barrier (BTB) model using vascular endothelial cells, miR-18a decreased OCL, CLDN-5, and ZO-1 mRNA expression acting on the runt-related transcriptional factor 1 (RUNX1) gene. This decreased mRNA translated to ZO-1, leading to increased permeability of BTB (Miao et al., 2015). Similarly, miR-34c was found to decrease OCL, CLDN-5, and ZO-1 abundance in an *In vitro* BTB model. This function of miR-34c is mediated by the Myc-associated zinc finger protein (MAZ). The *MAZ* gene is targeted by miR-34c, causing BTB disintegration and increased permeability (Zhao et al., 2015).

MicroRNAs are involved in glioma angiogenesis, a process entailing the formation of new blood vessels in a multistage manner involving endothelial cells, proteolytic enzymes, extracellular matrix components (ECM), and growth factors like VEGF, fibroblast growth factor (FGF), HIF1-α, and angiopoietins (Ang1, Ang2) (Zhang A. B. et al., 2023; Mafi et al., 2023). MicroRNA gene mutations and epigenetic alterations cause microRNA dysregulation in gliomas. While some microRNAs act as tumor suppressors, others play a role in carcinogenesis (Table 3).

**Table 3:** The effects of microRNAs on cancerogenesis in gliomas.

	Tumor suppressive miRNA	Cancerogenic miRNAs
Downregulated in gliomas	miR-7, miR-34a, miR-124, miR-128, miR-137, miR-181 (Banelli et al., 2017; Tluli et al., 2023; Silber et al., 2008) miR-7, miR-34a and miR-12 (Chen M. et al., 2021) miR-128 (Godlewski et al., 2008)	RAS inhibitor Let-7 (Lee et al., 2011)
Upregulated in gliomas	anti-tumor effect miR-128-3p or miR-145-5p (Goenka et al., 2022) inhibition of tumor-suppressing gene miR-19a, miR-19b, miR-20a, and miR-92a (Buruiană et al., 2020) inhibition of proapoptotic p53 MiR-221/222 (Chen et al., 2012)	miR-10b and miR-21 in high-grade gliomas have been linked to angiogenesis, metastasis, and chemoresistance (Chen M. et al., 2021) Upregulated in proneural (PN) but inactive in mesenchymal (MES) GBM miR-125b and miR-20b (Huang et al., 2016) miR-10b (El Fatimy et al., 2017) miR-21 3p, and miR-21-5p (Shang et al., 2015)

### 3.3 Glioblastoma programmed cell death effect on TJ

The progression of glioblastoma depends on the equilibrium between processes that maintain tumor cell survival and mechanisms that lead to programmed cell death (PCD). However, PCD is controversial in the treatment of glioblastoma since it stimulates immunosuppressive mechanisms, ultimately resulting in poor outcomes. Ferroptosis, an iron-dependent PCD mechanism observed in glioma, is associated with a gloomy prognosis due to tumor cell migration, temozolomide resistance (Liu et al., 2020), induced immunosuppression, and resistance to immunotherapy (Liu D. et al., 2022). An increase in PCD protein 10 (PDCD10) in glioblastoma cells prompts an increase in ZO-1 and CLDN-5 in co-cultured endothelial cells (Wu et al., 2023). The localization of ZO-1 within TJs is moderated by JAM-A, which also has an interactive relationship with ZO-1. Furthermore, JAM-A suppresses microglial activation surrounding glioblastoma in female mice (Turaga et al., 2020). ZO-1 adjusts transcription through its association with the Y-box binding protein 3 (YB-3 / ZO-1-associated nucleic acid-binding protein, ZONAB), influencing cell proliferation (Balda and Matter, 2000) and angiogenesis (El Bakkouri et al., 2024).

Tumor cells are shielded from apoptosis by the F11 receptor (F11R)/JAM-A (F11R/JAM-A), which is a member of the JAM family of TJ proteins. The abundance of F11R/JAM-A is magnified in glioblastoma and its heightened levels are associated with reduced patient survival and an overall unfavorable outcome (Lathia et al., 2014). Nevertheless, in the case of grade 2 and grade 3 gliomas, no relationship has been noted between the abundance of F11R/JAM-A and patient survival (Rosager et al., 2017).

### 3.4 TJ reorganization and peritumoral edema

The extent of TJ protein reorganization is associated with the grading of glial tumors. The abundance of OCL, CLDN-1, and CLDN-5 was observed to be reduced in anaplastic astrocytoma and glioblastoma (Ishihara et al., 2008). Conversely, CLDN-4 abundance was higher in glioblastoma than in low-grade tumors (Yan et al., 2022). OCL presence was found in 68.3% of brain tumors. Almost 80% of low-grade tumors were OCL-positive, while 25% of high-grade gliomas demonstrated OCL positivity. The highest OCL positivity was detected in hemangioblastomas (100%), pituitary adenomas (100%), and schwannomas (83.3%). The volume of peritumoral brain edema was lower in OCL-positive cases than in negative ones. Furthermore, the average survival time was longer in the OCL-positive tumor group than in the OCL-negative cases (Park et al., 2006). This study analyzed OCL content in tumor homogenates, hence, it does not offer information about the relocation of OCL or its presence in a subset of microvessels. Nonetheless, the study supports the use of OCL, and also CLDN-5, as prognostic biomarkers, which have already been used in other brain pathologies like stroke (Kazmierski et al., 2012), neuromyelitis optica (Jasiak-Zatońska et al., 2022) or fetal growth restriction syndrome with neuronal injury (Misan et al., 2022) in terms of BBB disintegration. The loss of OCL content in glial tumors was suggested as a contributing factor to endothelial TJ opening (Papadopoulos et al., 2001). The association between OCL content, peritumoral edema (PTBE), and survival time in brain tumor patients aligns with other reports that underscore the role of TJ proteins. CLDNs also play an essential role in maintaining BBB integrity. CLDN-5 is disintegrated in pathological conditions by matrix metalloproteinase-9 (MMP-9), which increases BBB permeability (Nitta et al., 2003). It is well-established that MMPs, along with aquaporin-4 (AP-4) and VEGF, contribute to PTBE. However, the involvement of TJ demands further research. CLDNs’ role in tumorigenesis has been extensively studied with a focus on peritumoral edema, specifically, employing a 3D spheroid BBB model and samples from patients with gliomas ranging from grade 1 to 4 (Abuelrub et al., 2024). In the model incorporating human umbilical vein endothelial cells (HUVEC) and glioma cells, *CLDN1* gene expression was augmented in *IDH1*-wildtype. In contrast, no significant *CLDN1* gene expression was observed in *IDH*-mutant cell lines (Abuelrub et al., 2024). Moreover, *CLDN3* and *CLDN5* gene expressions were escalated in both *IDH1*-wildtype and *IDH*-mutant cell lines (Abuelrub et al., 2024). In the same study, lower *CLDN1* gene expression was noted in patient samples with PTBE than in non-PTBE samples. In patient samples, both with PTBE and non-PTBE, no differences in *CLDN3* and *CLDN5* gene expression were found (Abuelrub et al., 2024). AQP-4 abundance in PTBE positively correlated with both VEGF and HIF-1α content (Mou et al., 2010). VEGF, derived from astrocytes, decreases OCL and CLDN-5 abundance in the endothelium (Argaw et al., 2009). VEGF rapidly enhances the phosphorylation of both OCL and the tyrosine of ZO-1 (Antonetti et al., 1999). High-grade astrocytomas produce VEGF more intensively (grade 3–67%, grade 4–64% vs. grade 2–37%), which stimulates angiogenesis, depletes OCL abundance, and enhances endothelial cell permeability (Oehring et al., 1999). VEGF provokes the redistribution of OCL from the endothelial cell membrane to intracellular endosomes. This process is mediated by OCL ubiquitination and phosphorylation induced by VEGF, which further leads to OCL degradation in the ubiquitin-proteasome system. Ubiquitination is sufficient to induce OCL endocytosis and the disruption of the TJ complex resulting in the loss of CLDN-5 and ZO-1 abundance in the cellular membrane (Murakami et al., 2009). Neuropilin-1 (Nrp1) is a VEGF co-receptor involved in angiogenesis in the tumor microenvironment. In human brain microvascular endothelial cells (HBMVECs), Nrp1 stimulates IFN-*γ*-mediated activation of signal transducer and activator of transcription 3 (STAT3) and CXCL10 through Rac1 signaling, leading to the disruption of BBB (Smith et al., 2022). Endothelial cells are surrounded by perivascular astrocyte processes (PAP), which are remodeled by growth/differentiation factor 15 (GDF15) that stimulates CLDN-5 abundance (Malik et al., 2020).

### 3.5 TJ proteins and glioblastoma treatment possibilities

The current efficacy of glioblastoma treatment is unsatisfactory, which makes research into tumor heterogeneity, glioma stem cells, DNA damage repair mechanisms, and various immunotherapies essential. These include therapies like immune checkpoint inhibitors, chimeric antigen receptor T (CAR T) cell therapy, oncolytic virotherapy, vaccine therapy, and agents interfering with tumor metabolism, along with strategies to overcome the BBB for effective drug delivery. Modification of TJ proteins plays a significant role in treatment strategies that focus on the BBB.

In the tumor microenvironment, interactions between tumor cells lead to cell adhesion-mediated drug resistance (CAM-DR). This resistance is observed in various tumors, including gliomas and glioblastomas (Westhoff et al., 2008; Ding et al., 2020). This process is mediated by integrin αV, which reduces EGFR abundance and stimulates the FAK (focal adhesion kinase)/paxillin/AKT (a serine/threonine protein kinase) pathway (Yu et al., 2021).

In an experimental glioma model, FAK was found crucial in destabilizing the tumor endothelium, evident by the tortuous, distorted, and hyperdilated vasculature (Lee et al., 2010). The presence of FAK resulted in reduced ZO-1 and OCL abundance and increased vascular permeability (Lee et al., 2010). The coupling of FAK to integrin αV is stimulated by VEGF (Avraham et al., 2003).

Currently, anti-FAK drugs are considered potential modifiers of the tumor microenvironment, and their synergistic effect with chemo- and immunotherapy promises improved drug resistance (Zhang J. et al., 2023). AKT inhibitor perifosine, when used with temozolomide, suppressed the viability and proliferation of glioblastoma cells through caspase-dependent apoptosis (Zhao et al., 2024b). In addition, perifosine, an alkylphospholipid analog, reversibly opens TJs (Koklic, 2014). To date, there are no reports on the impact of other AKT inhibitors such as AZD5363/capivasertib and MK-2206 on TJs.

Ibrutinib, a Burton tyrosine kinase (BTK) inhibitor, significantly reduces both the expression and abundance of OCL, CLDN-3, CLDN-5, ZO-1, tricellulin/MarvelD2 both at gene and protein levels 2 h after administration in a rat glioma model (Lim et al., 2024). This evidence about TJ proteins and BBB integrity and permeability in glioblastoma presents new directions for further research into combined therapies. The ultimate goal is to enhance drug penetration through BBB and impact signaling pathways crucial for tumor progression and aggressiveness, thereby improving therapeutic efficacy.

The depletion or modification of TJ proteins is hypothesized to facilitate drug delivery for tumor treatment. For instance, lipid polymeric nanoparticles (LPN) may disrupt CLDN-5 and ZO-1, thus enhancing paracellular transport and permeability, and subsequently promoting afatinib delivery across a BBB model (Lo et al., 2021). Another strategy to facilitate drug delivery across the BBB involves exploiting the transcytosis of the transferrin receptor (TfR) (Esparza et al., 2023). TfR abundance is significantly elevated on the endothelial cells of glioma tumor vasculature, making it a promising avenue for glioma therapy. Notably, targeting TfR with vincristine-carrying liposomes has led to effective BBB penetration of the drug and significant anti-glioma therapeutic effects (Mojarad-Jabali et al., 2022).

In contrast, TJ stabilization is crucial for the management of peritumoral edema. The anti-diabetic sulfonylurea derivative, glyburide, generates this effect by reducing the gaps among ZO-1 proteins along the cell membrane, effectively decreasing BBB permeability (Thompson et al., 2013).

## 4 Conclusion

This review emphasizes the roles and attributes of TJ proteins in glial tumors. These proteins’ participation in various processes such as intercellular adhesion, proliferation, neoangiogenesis, and invasiveness suggests their crucial role in both the pathogenesis of glial tumors and their clinical course. The disorganization and perturbed formation of the TJs in glioma vessels induce BBB disruption and subsequent brain edema development. Numerous factors, which include cytokines and miRNAs, alter the abundance of TJ proteins. These proteins’ functions are regulated through phosphorylation/dephosphorylation, ubiquitination, and palmitoylation. Occludin, claudins, and ZO-1 represent key TJ proteins. Enhancing understanding of their regulation in glial tumors could amplify the quality of care for patients with gliomas. Future investigations concentrating on TJ proteins in liquid biopsies might yield diagnostic protocols contributing to the early detection of glial tumors and monitoring of post-surgical treatment patients. Deciphering their precise contributions to glioma pathomechanisms may pave the way for novel therapeutic approaches.
